# Navigating Boundaries in Female Psychiatric Intensive Care Units: Risk, Autonomy and Trauma-Informed Practice Through a Gender Sensitive Lens

**DOI:** 10.1192/bjo.2026.11877

**Published:** 2026-06-30

**Authors:** Krishna Prakash

**Affiliations:** SHPU NHS Trust, Sheffield, India

## Abstract

**Aims:**

Background

Women admitted to Psychiatric Intensive Care Units (PICU) frequently present with complex trauma histories, emotional dysregulation and heightened sensitivity to coercion, confinement and interpersonal authority. While restrictive boundaries may be required to maintain safety, poorly implemented or inflexible restrictions risk re-traumatisation, escalation of distress and disruption of therapeutic engagement. There is limited literature exploring how boundary implementation itself influences safety and engagement for women in PICU.

Aim

To explore how boundary-related clinical decisions influenced safety, emotional regulation and engagement in women admitted to PICU, and to identify gender-sensitive, trauma-informed approaches perceived by clinicians as helpful in practice.

**Methods:**

Methods

A qualitative service evaluation using reflective thematic analysis was undertaken involving three anonymised female PICU admissions detained under the Mental Health Act. Cases were purposively selected to illustrate differing trajectories following boundary implementation: escalation, de-escalation and neutral outcomes. Data were derived from contemporaneous clinical documentation and multidisciplinary team reflections. The evaluation aimed to generate practice-based learning rather than generalisable outcomes.

Direct service-user interviews were not conducted to avoid placing additional demands on patients during acute admission. Findings reflect clinician interpretations of observed patterns of response to boundary setting.

**Results:**

Results

Three key patterns emerged:

Collaborative and relational boundary setting was associated with reduced agitation and improved engagement.

Blanket or rigid restrictions were more frequently linked with trauma re-experiencing, protest behaviours and escalation.

Necessary restrictive interventions, when poorly communicated, negatively affected dignity and delayed therapeutic re-engagement, even when clinically justified.

Gender-sensitive negotiation, preservation of autonomy where safe, and compassionate communication appeared clinically impactful.

Discussion

The findings suggest that in female PICU populations, how boundaries are enacted may be as influential as what restrictions are applied. Trauma-informed, collaborative approaches may mitigate escalation while maintaining safety. A reflective boundary-decision framework is proposed to support proportionality, transparency and restoration of autonomy.

**Conclusion:**

Conclusion

Boundary implementation in female PICU settings has important implications for safety, emotional regulation and therapeutic alliance. Gender-sensitive, trauma-informed boundary practices may support safer care and improved engagement. Future work should incorporate co-produced evaluation and service-user perspectives to strengthen the evidence base.

